# Full-thickness defect closure using the reopenable clip over-the-line method with omental patch

**DOI:** 10.1055/a-2133-6266

**Published:** 2023-08-21

**Authors:** Tatsuma Nomura, Shinya Sugimoto, Yu Fujimura, Keiichi Ito, Yasuo Katsumine, Noriya Uedo

**Affiliations:** 1 Department of Gastroenterology, Ise Red Cross Hospital, Ise, Mie, Japan; 2 Department of Gastroenterology, Mie Prefectural Shima Hospital, Shima, Mie, Japan; 3 Department of Surgery, Mie Prefectural Shima Hospital, Shima, Mie, Japan; 4 Department of Gastrointestinal Oncology, Osaka International Cancer Institute, Osaka, Japan

Various endoscopic defect closure methods following endoscopic full-thickness resection (EFTR) of submucosal tumors have been developed 1 2 ; however, there is no established endoscopic defect closure method that can close a full-thickness defect as reliably as surgical suturing. We developed the reopenable clip-over-line method (ROLM), capable of closure of large mucosal defects and defect closure post-EFTR 3 4 . Here, we report the use of the ROLM with an omental patch (ROLM-OP), which includes closure of the serosal muscle layer using omental fat ( Video 1 ).

**Video 1** Gastric full-thickness defect closure using a reopenable-clip over-the-line method with an omental patch.


The patient had a 26-mm submucosal tumor on the anterior side of the antrum that was endoscopically resected via full-thickness resection with laparoscopic assistance ( Fig. 1 ). The diameter of the full-thickness defect was approximately 30 mm and ROLM-OP was used to achieve complete defect closure. First, a clip with line was placed to grasp the serosal muscle layer and mucosa on the anal side. Next, a reopenable clip with a line through the tooth hole on one side was placed to grasp the serosal muscle layer and the mucosa of the contralateral defect edge. By repeating this procedure, the bilateral defect edges were gradually closed. As the omental fat was endoscopically visible, the reopenable clip could grasp the omental fat, serosal muscle layer, and mucosa. Laparoscopy confirmed that the omental fat was inserted in the closure line, and the full-thickness defect was completely closed using ROLM-OP. A negative laparoscopic leak test result confirmed complete closure, and the procedure was therefore completed without additional suturing.

**Fig. 1:**
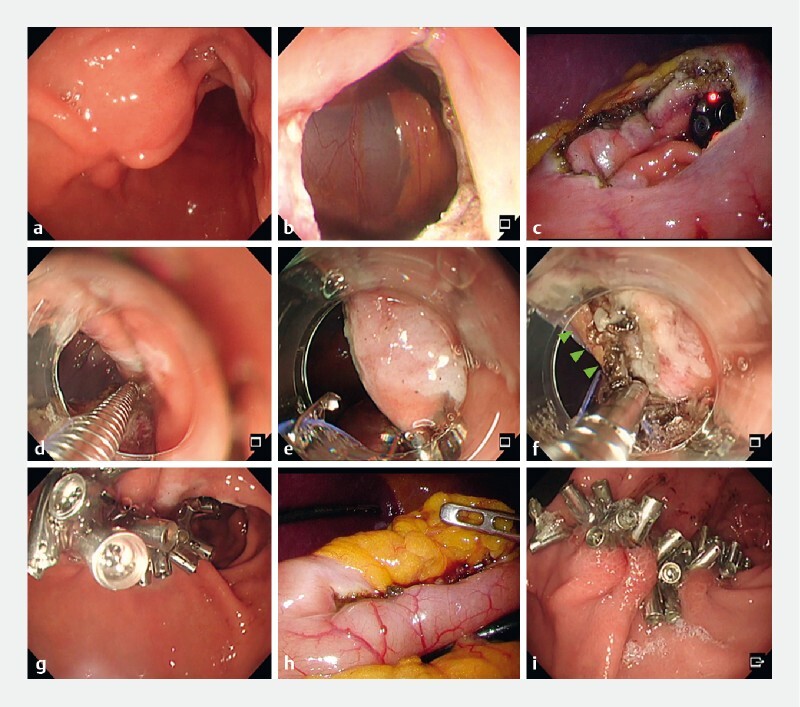
Images from the reopenable clip-over-line method with an omental patch (ROLM-OP) for defect closure after endoscopic full-thickness resection (EFTR) showing: **a** a 26-mm gastrointestinal stromal tumor (GIST) on the anterior side of the antrum; **b, c** the full-thickness defect after EFTR, which was 30 mm in diameter; **d** the first clip with line placed to grasp both the serosal muscle layer and the mucosa distal to the defect; **e** the next reopenable clip, with a line in hand threaded through the tooth on one side of it, placed to grasp the contralateral serosal muscle layer and mucosa of the defect; **f** fatty tissue in the abdominal cavity (green arrows) after repeated ROLM procedures, with the reopenable clip placed to grasp both the serosal muscularis and fatty tissue (ROLM-OP); **g** the completely closed full-thickness defect, which had a negative leak test result, from the endoscopic side; **h** the completely closed mucosal defect on laparoscopic view; **i** the completely closed mucosal defect 7 days after EFTR and endoscopic closure, with the clips still in place.

Fluoroscopy, at 3 days post procedure, revealed no leakage, and the patient was allowed a liquid diet. Endoscopic follow-up 7 days later showed all clips still in place and complete closure of the full-thickness defect. The patient was discharged without experiencing any adverse events. Therefore, ROLM-OP appears to be a novel and feasible technique for full-thickness defect closure.

Endoscopy_UCTN_Code_TTT_1AO_2AG

## Notes

Endoscopy E-Videos

https://eref.thieme.de/e-videos
*E-Videos* is an open access online section of the journal *Endoscopy* , reporting on interesting cases and new techniques in gastroenterological endoscopy. All papers include a high-quality video and are published with a Creative Commons CC-BY license. Endoscopy E-Videos qualify for HINARI discounts and waivers and eligibility is automatically checked during the submission process. We grant 100% waivers to articles whose corresponding authors are based in Group A countries and 50% waivers to those who are based in Group B countries as classified by Research4Life (see: https://www.research4life.org/access/eligibility/ ). This section has its own submission website at https://mc.manuscriptcentral.com/e-videos
